# Gene expression profiling of monkeypox virus-infected cells reveals novel interfaces for host-virus interactions

**DOI:** 10.1186/1743-422X-7-173

**Published:** 2010-07-28

**Authors:** Abdulnaser Alkhalil, Rasha Hammamieh, Justin Hardick, Mohamed Ait Ichou, Marti Jett, Sofi Ibrahim

**Affiliations:** 1 United States Army Medical Research Institute of Infectious Diseases, Fort Detrick, Maryland 21702, USA; 2 Division of Pathology, Walter Reed Army Institute of Research, Silver Spring, MD, 20910, USA

## Abstract

Monkeypox virus (MPV) is a zoonotic Orthopoxvirus and a potential biothreat agent that causes human disease with varying morbidity and mortality. Members of the Orthopoxvirus genus have been shown to suppress antiviral cell defenses, exploit host cell machinery, and delay infection-induced cell death. However, a comprehensive study of all host genes and virus-targeted host networks during infection is lacking. To better understand viral strategies adopted in manipulating routine host biology on global scale, we investigated the effect of MPV infection on Macaca mulatta kidney epithelial cells (MK2) using GeneChip rhesus macaque genome microarrays. Functional analysis of genes differentially expressed at 3 and 7 hours post infection showed distinctive regulation of canonical pathways and networks. While the majority of modulated histone-encoding genes exhibited sharp copy number increases, many of its transcription regulators were substantially suppressed; suggesting involvement of unknown viral factors in host histone expression. In agreement with known viral dependence on actin in motility, egress, and infection of adjacent cells, our results showed extensive regulation of genes usually involved in controlling actin expression dynamics. Similarly, a substantial ratio of genes contributing to cell cycle checkpoints exhibited concerted regulation that favors cell cycle progression in G1, S, G2 phases, but arrest cells in G2 phase and inhibits entry into mitosis. Moreover, the data showed that large number of infection-regulated genes is involved in molecular mechanisms characteristic of cancer canonical pathways. Interestingly, ten ion channels and transporters showed progressive suppression during the course of infection. Although the outcome of this unusual channel expression on cell osmotic homeostasis remains unknown, instability of cell osmotic balance and membrane potential has been implicated in intracellular pathogens egress. Our results highlight the role of histones, actin, cell cycle regulators, and ion channels in MPV infection, and propose these host functions as attractive research focal points in identifying novel drug intervention sites.

## Introduction

Monkeypox virus is a double-stranded DNA virus and one of the human pathogenic orthopoxviruses that include *Variola *(VARV), cowpox (CPX), and *Vaccinia *(VACV) viruses. The virus causes a disease that manifests similarly to smallpox, but with milder morbidity and lower mortality rates [1]. Variation in MPV virulence has been observed and mapped to defined geographic origins, e.g., virus isolates from Central Africa are more virulent than those from Western Africa [2,3]

Recent advances in molecular biology and genomics have improved our understanding of viral infection and replication mechanisms. Monkeypox virus has a relatively large genome of about 196,858 base pairs, encoding 190 open reading frames, which constitute the bulk of the material needed for viral replication in cell cytoplasm [4]. Viral entry into cells is dependent on cell type and viral strain, and occur after an initial attachment to cell surface through interactions between multiple viral ligands and cell surface receptors [5] such as chondroitin sulfate [6] or heparan sulfate [7,8]. Subsequent crossing of cell membrane is mediated by a viral fusion event with cell membrane under neutral pH conditions [9], or by endosomal uptake via a macropinocytosis-like mechanism that involves actin [10,11] and low pH-dependent steps [12]. Once in the cell cytoplasm, the virus releases prepackaged viral proteins and enzymatic factors that disable cell defenses and stimulate expression of early genes [13-15]. Synthesis of early proteins promotes further uncoating, DNA replication, and production of intermediate transcription factors. In following stage, intermediate genes are transcribed and translated to induce the expression of late genes that function mainly as structural proteins, enzymes, and early transcription factors. Eventually, membrane structures will appear and unit virion genomes processed from DNA concatemers are assembled into nascent virions that contain all enzymes, factors, and genetic information needed for a new infectious cycle.

The detailed available information about viral gene functions and its programmed expression during infection exceeds current knowledge of corresponding events in the host. Furthermore, although poxviruses are considered one of the most self-sufficient viral families, they remain unable to reproduce in extracellular environment and known to have limited host range, which suggest dependence on host elements [16,17]. Therefore, identification of these specific host elements and pathways that are essential for viral replication will enrich our knowledge of host response to viral infection, and may prove valuable in identifying potential targets for antiviral therapies.

Microarrays have been used in genome exploration and profiling with special focus on understanding dynamics of viral gene expression and pathogenesis [18,19]. However, a paucity of work employed this tool in examining host response to infections with poxviruses generally [20-22], and more specifically in the case of MPV. Because combining microarray technology with modern data mining tools allows further information extraction at genome-wide levels, we used whole genome rhesus macaque microarrays in combination with Ingenuity Pathways Analysis (Ingenuity^® ^Systems, http://www.ingenuity.com) to investigate the effect of MPV infection on host *Maccaca mulata *kidney epithelial cells transcriptome, and address gaps in host response during MPV infection. Functional and canonical pathway analysis of differentially expressed genes at 3 and 7 hours post-infection (hpi) time points validated many of the known host gene responses to poxvirus infection and introduced new sets of interesting functions and pathways in areas of cell death and apoptosis, actin dynamics, ion channels and transport, and cell cycle regulation. Our data points to a vital role for these cell functions in MPV infection, and hence, signify their value in poxviruses' infection diagnosis and treatment studies.

## Materials and methods

### Cell culture and viral infection

Monkeypox virus-Katako Kombe strain (MPV-KK) was propagated in Vero E6 cells maintained in Eagle's Minimum Essential Medium with non-essential amino acids (EMEM/NEAA) supplemented with 2 mM L-glutamine, 10% heat-inactivated fetal bovine serum (FBS), 10 mg/L Gentamycin, 250 μg/L Fungizone, and buffered at pH 7.4 with 10 mM HEPES [23]. Viral titers were determined by the plaque assay [24]. As described previously [25], monolayer of Vero E6 were inoculated with serial dilutions of viral suspension and allowed to adsorb for 30 min at room temperature. The viral inoculate was removed and cells were washed twice with PBS then incubated with culture medium for 5 days. To count plaques, culture medium was removed and cells were fixed and stained simultaneously using 30% formalin, 5% ethanol (vol/vol) solution containing 1.3 g/L crystal violet.

For time points and control samples, MK2 cells were grown in the same culture medium described above but in absence of antibiotics and Fungizon for at least 2 days before infection with MPV. Culture medium was removed and cells were inoculated with crude monkeypox virus-Katako Kombe strain (MPV-KK) at MOI of 3. Virus was adsorbed for 30 min at 37°C with gentle rocking for 15 sec each 10 min, then cells were washed twice with room temperature equilibrated PBS, fresh culture medium was added, and cells were incubated for 3 or 7 hours. Control cells were handled identically except for exposure to virus.

### RNA and cDNA preparation, labeling, hybridization, and scanning

RNA was extracted using TRIzol LS (Invitrogen) according to the manufacturer's recommended protocol. Briefly, 200 μl of chloroform (Sigma Aldrich, St. Louis, MI) was added to 5×10^6 ^infected and non-infected control cells harvested in 1 ml of TRIzol LS reagent (Invitrogen). Solutions were mixed thoroughly and incubated for 10-15 min at 4°C, then centrifuged for 20 min at 14,000 rpm in 4°C. After centrifugation, the aqueous phase was removed, added to an equal volume of 2-propanol (Sigma-Aldrich). After overnight incubation at -20°C, the mixture was centrifuged for 20 min at 14,000 rpm in 4°C. Supernatant was removed and the RNA pellet was washed with 80% ethanol (Sigma Aldrich), dried, and dissolved in 100 μl of RNase-free water (Ambion, Austin, TX). Possibly present contaminating DNA was eliminated using Turbo DNA-free kit (Ambion), and RNA clean up was performed using RNeasy kit (Qiagen, Valencia, CA). Quality and quantity of RNA was evaluated using Experion automated electrophoresis station and Experion RNA StdSens analysis kit (Bio-Rad, Hercules, CA). Clean sharp peaks representing intact rRNA were confirmed for each preparation by two independent workers.

cDNA was synthesized using One-cycle cDNA Synthesis kit (Affymetrix, Santa Clara, CA) in presence of poly-A RNA controls. Double-stranded cDNA samples were cleaned up and biotin labeling of antisense cRNA was carried out with the IVT Labeling Kit (Affymetrix, P/N 901229). Material was cleaned up then fragmented before hybridization overnight. Microarrays (Affymetrix, P/N 900656) were scanned using the Affymetrix GeneChip scanner following standard Affymetrix protocols [26] after carrying out all washing and staining steps as recommended by the manufacturer.

### Microarray validation by RT PCR

RT PCR was performed utilizing the Superarray human common cytokines panel (PAHS-021E-4, Superarray, Frederick MD, 21703). Reactions were performed according to manufacturer's guidelines for both time points in 10 μl volumes using the 384 plate format that allow gene expression analysis in quadruplicate. Results showed a strong correlation of gene expression levels with that obtained using microarray.

### Data analysis

The Affymetrix CEL files were imported into GeneSpring GX software v 7.3.1 (Agilent), which allows multi-filter comparisons using data from different experiments, to perform the normalization, generation of restriction lists and functional classifications of the differentially expressed genes.

Normalization was applied in two steps: i) "per chip normalization" by which each measurement was divided by the 50th percentile of all measurements in its array; and ii) "per gene normalization" by which each treated sample was normalized against its respective control (mock-treated) sample.

The expression of each gene was reported as the ratio of the value obtained relative to the control condition after normalization of the data. Transcripts whose levels reproducibly changed were identified using one-way parametric analysis of variance with a P-value cutoff of 0.05 (false discovery rate of 5%). The changes in transcript levels are expressed as the fold change in signal between control and treated samples.

Two-dimensional clustering was carried out based on samples and genes for visualization and assessment of reproducibility in the profile of the significant genes across biological replicates.

## Results

### Dataset overview

To characterize and measure changes in transcriptome of cells infected with MPV, we infected 10^7 ^*Macaca mulatta *kidney epithelial cells (MK2) and incubated them for 3 or 7 hpi. Cells were infected at high multiplicity to enhance infection probability and improve signal to noise ratio. Infected cells were washed after virus adsorption to reduce response to any biological factors in the seed, and to avoid an infection continuum that may desynchronize virus replication stages. We selected the two time points not to exceed 7 hpi to avoid possible entry into a second cycle of viral replication, and to minimize artifacts that might arise from cell lysis after viral maturation and release of intracellular components [18]. We mock-infected a similar number of cells and used them as controls for each time point. The experiment was done in triplicate, and 47,000 rhesus macaque transcripts potentially present in purified RNA from each sample were interrogated by 52,000 probe sets/microarray.

**Figure 1:**
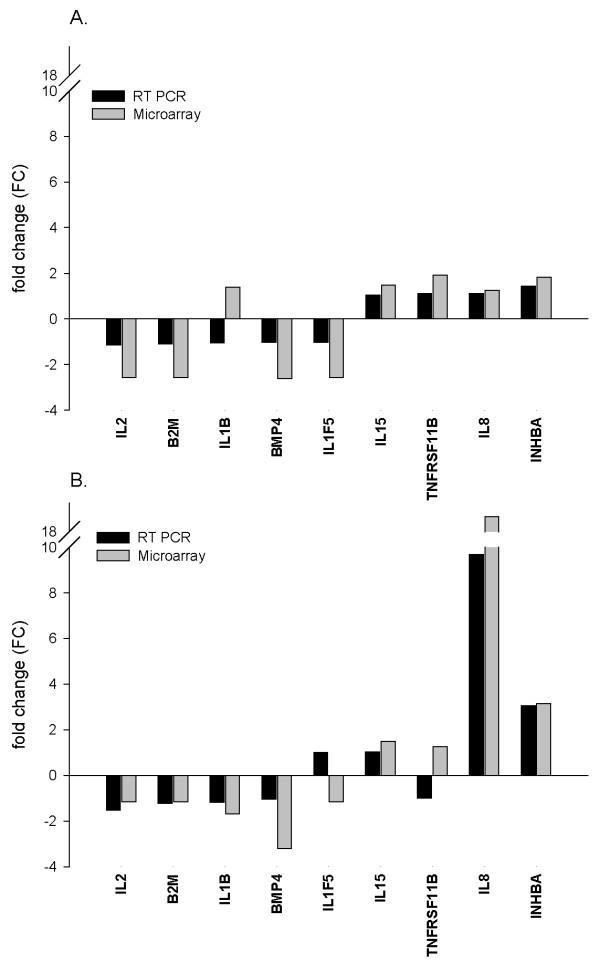
**Validation of microarray results by quantitative real-time PCR**. Copy number of eight common cytokines and a house keeping gene (B2M) were calculated based on RT-PCR Ct values. Fold change of genes expression is plotted on Y-axis after normalization to mock-treated samples. Results plotted to compare calculated fold change in expression of each gene at 3 hpi (A) or at 7 hpi (B) using Microarray (grey bars) and RT-PCR (black bars).

We employed RT-PCR as a tool to validate microarray results. Expression levels of nine genes encoding eight cytokines and one housekeeping protein were assessed and the resulting fold change for each gene was compared with corresponding calculated measurements from the microarray study. The RT-PCR measurements were carried out in quadruplicate, and cycle threshold (Ct) values in each observation were normalized to the average of five housekeeping genes and to the expression level of the same gene in control mock-infected cells as described in microarray experiments. Despite consistent mild lower fold change values in all assessed genes by RT-PCR, a direct correlation was evident between the results obtained in both techniques, and 15 out of the 18 gene expression measurements in 3 hpi (Fig. 1A) and 7 hpi (Fig. 1B) were concordant. The moderately staggered prediction in gene regulation in the other three measurements coincided with large Ct values, which were near or beyond RT-PCR detection sensitivity limitations. Likewise, the observed lower and consistent fold change FC estimates from RT-PCR where mainly due to different normalization methods used for each approach. The strong correlation in results of RT-PCR and microarray was mutually validating and instigated subsequent data analysis.

### MPV compromises host's biological activities with a dominating global downregulation

**Figure 2:**
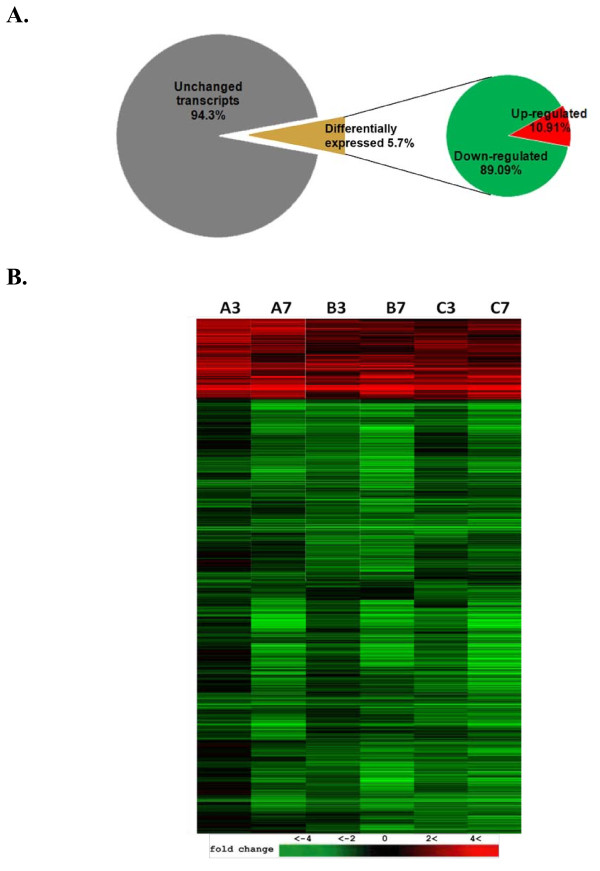
**(A) Gene expression overview of *Macaca mulatta *kidney epithelial cells infected with MPV**. (A) Using one-way ANOVA, a total of 2702 elements exhibited reproducible change (P-value ≤ 0.05). This represented 5.7% of the total 47000 interrogated transcripts on the GenChip. About 89% of the differentially expressed transcripts were suppressed and less than 11% were up-regulated. (B) Expression data for significantly influenced transcripts were hierarchically clustered. Columns represent triplicate (A, B, C) of time points 3 and 7 hpi. Color intensity reflects fold change relative to control (mock-transfected) cells. Red and green indicate up- and downregulation respectively.

Downregulation was reported as the hallmark of gene expression modulation in *Vaccinia*-infected human HeLa cells [20] and in lymphocytes of *Variola*-infected cynomolgus macaques (*Macaca fascicularis*) [21]. In this study, we identified 2,702 transcripts that exhibited statistically significant changes in copy numbers with P values < 0.05. This represents about 5.7% of the total interrogated host transcripts on the microarray (Fig. 2A). In agreement with previously reported results [21,22], the majority of transcription changes we observed were downregulation. More than 89% of the regulated genes or 2,407 genes exhibited steady downregulation in both 3 and 7 hpi time points, while only 295 genes or 10.92% of total regulated gene showed upregulation under the same statistical constraints (Fig. 2B).

### Up- and down-regulated genes exhibit varied temporal regulation distribution

**Figure 3:**
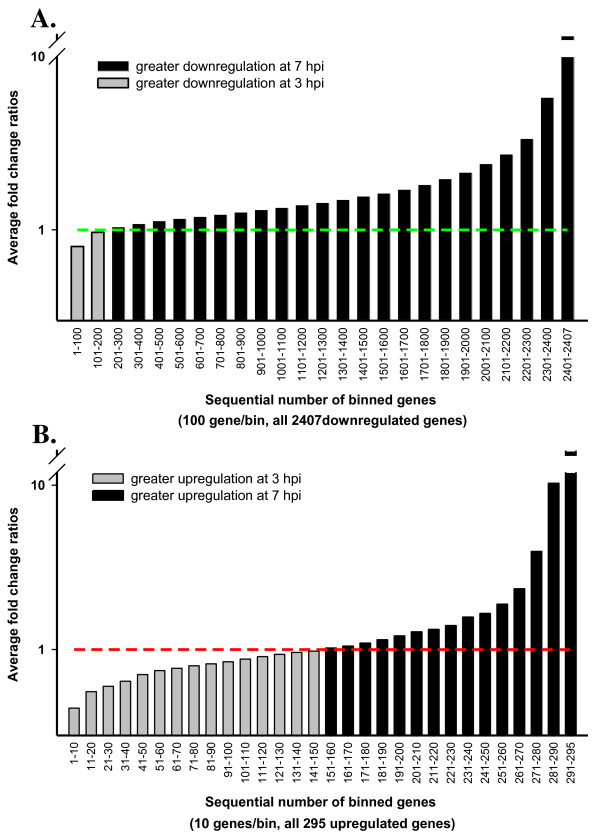
**Up- and down-regulated transcripts follow different distribution patterns during infection progression**. Each bar represents average fold-change ratios (FCRs) of gene expression at 7 hpi/3 hpi for either 100 consecutive downregulated genes (A), or 10 upregulated genes (B), after sorting all genes according to their FCR values from smallest to largest. Genes with average FCR values < 1 are in grey and those with values > 1 are in black. Results show most downregulated genes maintained an increasing suppression trend in the course of infection as more than 92% of the genes gave fold-change ratio > 1 (A). The 295 upregulated genes exhibited more balanced distribution between 7 and 3 hpi with about 51% of the genes having higher fold change at early time point.

Because MPV exhibit well-defined temporal gene expression stages that were classified into early, intermediate, and late stages, we anticipated the host response to exhibit some sort of an analogous pattern. This may be represented in variable temporal expression of regulated host genes, where a subset will exhibit a higher copy number at early time point followed by a decline at late time point as the inducing viral protein or gene decreases or disappears. We followed peaks of gene expression regulation for each of the differentially expressed genes across both time points by dividing gene expression fold change (FC) at late time point (7 hpi) by FC of the same gene in early time point (3 hpi). Calculated fold change ratios (FCRs) 7/3 hpi for all 2,407 down-regulated genes are plotted in (Fig. 3A). Bars represent the average FCR for each consecutive 100 genes after sorting them according to their FCR values from smallest to highest for data clarity. A plot for all 295 upregulated genes was made following identical steps (Fig. 3B), but bars represent the average FCR of 10 sequential genes. Genes with FCR > 1 indicate a relatively greater regulation in later stages, while < 1 ratio points to greater regulation in early stage. Only 7.9% of the downregulated genes showed higher fold change early in the course of infection (Fig. 3A), while 51.1% of the upregulated genes exhibited similar temporal expression trend (Fig. 3B). In contrast with downregulated genes (Fig. 3A), peaks of host gene upregulation were almost equally distributed across infection time points (Fig. 3B). This important difference in the distribution of temporal up and downregulated genes is a new landmark of global host gene regulation by poxviruses second to known dominant host genes downregulation [20-22]. While the steady increase in host gene suppression denotes known continuous viral stifling of host functions such as innate immune response and antiviral cell defense mechanisms, the variation in host gene upregulation intensity seems to mark the stage dependent viral gene expression and need for specific host material or machinery.

### Functional gene clusters

**Figure 4:**
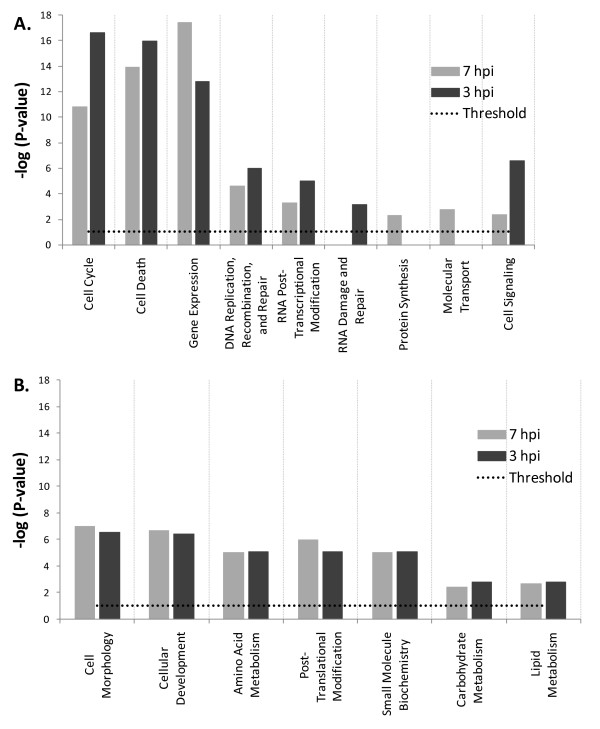
**Comparative functional analysis of differentially expressed genes at 3 and 7 hpi**. **(A) **Represents functions exhibiting -log (P-values) differences ≥ 1.301 between 3 and 7 hpi time points. Few identified functions were unique to one time point only under adopted statistical constraints. **(B) **Equally present functions in both time points showing a -log (P-value) differences ≤ 1.301. All functions were identified with P-values ≤ 0.05 or -log (P-value) = 1.301 thresholds (dotted black lines).

We used Ingenuity pathway analysis (IPA) to identify the main host functions and canonical pathways influenced by viral infection. The differentially expressed genes at 3, 7 hpi time points fell into diverse functional categories including enzymes, transcription regulators, kinases, phosphotases, peptidases, transmembrane receptors and G protein coupled receptors, transporters, translation regulators, and micro RNAs (Table. 1). To reduce noise in functional analysis, we applied a second data filter and included only genes that exhibited ≥ 1.8 FC in addition to the t-test filter with P values < 0.05 already in place. A total of 1,013 and 1,720 genes from 3 and 7 hpi time points, respectively, met analysis thresholds and were used in a comparative functional analysis to identify unique and common influenced host functions for both time points. Results were scored based on Fisher's exact test, and functions with P-values ≤ 0.05 were considered statistically significant. When defined the -log (P-value) ≥1.301 as a significant difference in random genes clustering probability for a given function between the two time points, functions clearly separated into either time point dependent or independent categories (Fig. 4). Functions relating to cell signaling, cell cycle, cell death, transcriptional modification, and DNA processing were more significant in 3 hpi time point (Fig. 4A), and while protein synthesis and molecular transport functions were indentified with significant P-values only at 7 hpi, and RNA damage and repair was found to be unique to 3 hpi time point (Fig. 4A). Time-point independent functions exhibited comparable -log (P-value) in both time points and included metabolism of essential building blocks such as amino acids, lipids, and carbohydrates (Fig. 4B), and other functions related to cell morphology, cellular development, small molecule biochemistry, and posttranslational modification (Fig. 4B).

**Table 1:** Functional distribution of differentially expressed genes at 3, 7 hpi time points

Gene function category	Number of genes	Up regulated		Down regulated		FC < 1.8	
		
		3 hpi	7 hpi	3 hpi	7 hpi	3 hpi	7 hpi
transporter	122	10	8	43	88	69	26
transmembrane receptor	19	3	3	5	13	11	3
translation regulator	16	5	5	5	9	6	2
transcription regulator	261	12	12	107	197	142	52
phosphatase	48	2	1	17	33	29	14
peptidase	48	4	4	9	27	35	17
other	1278	128	104	452	852	697	322
microRNA	1	-	-	1	1	0	0
ligand-dependent nuclear receptor	7	-	-	1	4	6	3
kinase	134	8	4	58	96	68	34
ion channel	11	-	-	5	9	6	2
growth factor	8	1	1	4	4	3	3
G-protein coupled receptor	13	1	2	8	10	3	3
enzyme	308	32	31	89	199	187	78
cytokine	5	2	2	1	1	2	2

The expression of histones exhibited interesting pattern with potentially important implications in viral replication. Functional analysis showed steep upregulation of histones in both time points with sharp increase through the course of MPV infection. Except for HIST3H2A, all other core histone genes including HIST1H3I, HIST1H1D, HIST1H2BJ, HIST1H2AD, and HIST4H4 exhibited upregulation (Table. 2). The linker histone H1F0, was downregulated with an increasing trend. Unlike the vast increase in histones expression, genes encoding major transcription regulators of histones expression such as CITED2, NCOA3, CREB1, YY1, and HDAC2 showed increasing suppression throughout infection. Similarly, many enzymes controlling major modifications of histones and chromatin organization dynamics of the host, including FBXO11, PRMT3, MYST2, MYCBP2, and RARS2 showed steady downregulation (Table. 2).

**Table 2:** List of differentially expressed genes involved in histones dynamics in MPV- infected cells

Symbol	Type	Fold Change		Entrez Gene Name
			
		3 h	7 h	
FBXO11	enzyme	-1.98	-2.95	F-box protein 11
PRMT3	enzyme	-1.81	-3.04	protein arginine methyltransferase 3
MYST2	enzyme	-1.69	-3.73	MYST histone acetyltransferase 2
MYCBP2	enzyme	-1.66	-2.01	MYC binding protein 2
RARS2	enzyme	-1.41	-1.61	arginyl-tRNA synthetase 2, mitochondrial
CITED2^α^	transcription regulator	-8.75	-309.73	Cbp/p300-interacting transactivator, with Glu/Asp-rich carboxy-terminal domain, 2
NCOA3^α^	transcription regulator	-2.29	-4.76	nuclear receptor coactivator 3
CREB1^α^	transcription regulator	-1.91	-2.59	cAMP responsive element binding protein 1
YY1^α^	transcription regulator	-1.58	-2.60	YY1 transcription factor
HDAC2	transcription regulator	-1.38	-1.57	histone deacetylase 2
HIST3H2A	histone	-2.49	-9.19	histone cluster 3, H2a
H1F0	histone	-2.45	-6.15	H1 histone family, member 0
HIST1H3I	histone	2.64	22.09	histone cluster 1, H3i
HIST1H1D	histone	7.49	42.62	histone cluster 1, H1d
HIST1H2BJ	histone	16.57	160.56	histone cluster 1, H2bj
HIST1H2AD^α^	histone	46.04	994.02	histone cluster 1, H2ad,1
HIST4H4^α^	histone	1964.01	2989.9	histone cluster 4, H4

α Average gene expression values is presented for genes with multiple identifiers.

Another set of genes encoding mainly transmembrane proteins that function as ion channels or transporters were progressively suppressed during MPV infection. Out of 10 identified channels that exhibited statistically significant downregulation in the both data sets, eight were downregulated at 1.8-fold change or more (Table. 3). The majority of the identified channels were localized to the plasma membrane and only two had unknown locations in the cell. Identified infection-regulated channels covered the transport of the principal ions Na^+^, K^+^, Cl^-^, and Ca^++ ^which function in a concerted manner to define transmembrane potential and maintain cellular osmotic balance.

**Table 3:** List of MK2 cell genes exhibited differential expression upon infection with MPV and encoded ion channels or related proteins

Symbol	Type	Location	Fold Change		Entrez Gene Name
				
			3 h	7 h	
CLCN3 ^α^	ion channel	Plasma Membrane	-2.02	-6.68	chloride channel 3
KCNMA1	ion channel	Plasma Membrane	-2.11	-3.94	potassium large conductance calcium-activated channel, subfamily M, alpha member 1
MST150	ion channel	Unknown	-2.00	-3.63	MSTP150
KCMF1	enzyme	Unknown	-1.77	-2.81	potassium channel modulatory factor 1
SCNN1A	ion channel	Plasma Membrane	-1.62	-2.68	sodium channel, nonvoltage-gated 1 alpha
CUL5	ion channel	Cytoplasm, Nucleus	-1.69	-2.51	cullin 5
KCTD20	ion channel	Unknown	-1.24	-1.92	potassium channel tetramerisation domain containing 20
TRPC1	ion channel	Plasma Membrane	-1.87	-1.87	transient receptor potential cation channel, subfamily C, member 1
CACNB3	ion channel	Plasma Membrane	-1.31	-1.83	calcium channel, voltage-dependent, beta 3 subunit
SCLT1	transporter	Plasma Membrane	-1.62	-1.64	sodium channel and clathrin linker 1
TPCN1	ion channel	Plasma Membrane	-1.34	-1.60	two pore segment channel 1

α Average gene expression values are presented for genes with multiple identifiers.

### Canonical pathways analysis

To identify the effect of viral infection on major biological processes in the cell, we examined potential enrichment of all differentially expressed genes in known canonical pathways. The two data sets were analyzed using IPA library of canonical pathways. Genes exhibited ≥ 1.8 FC and associated with a canonical pathway in the Ingenuity pathways knowledge database were considered for the analysis. Both data sets contained 2,702 genes from which 2,281 genes had mapped identities. The number of genes eligible for pathway analysis was 680 and 1,134 genes for time point 3 and 7 hpi, respectively. The significance of the association between a subset of genes in data sets and the canonical pathway was measured in two ways: 1) ratio of the number of genes from the data set that mapped to the pathway divided by the total number of genes that mapped to the canonical pathway, and 2) Fischer's exact test P-value, which determines the probability of random association between the genes in the datasets and the canonical pathway.

**Figure 5:**
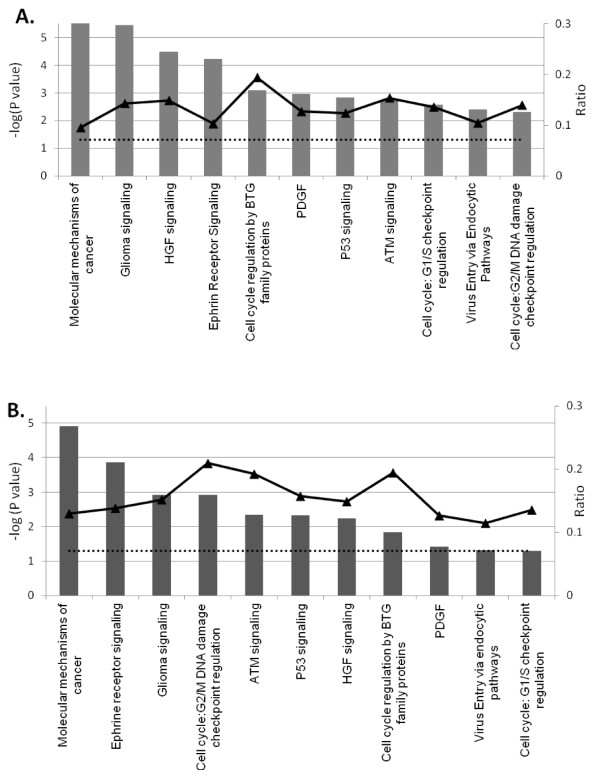
**Major influenced canonical pathways of MPV-infected MK2 cells**. Relevant pathways are shown at 3 (A) and 7 (B) hpi. The primary Y-axis shows the -log (P-value) of the probability for genes in data set to associate with identified pathway by chance. A threshold P-value of 0.05 or a -log (P) = 1.3 is presented in dotted line. The ratio of the number of genes from the data set that map to given pathway divided by the total number of genes that map to the canonical pathway is shown in solid line. All enlisted pathways met ratios ≥ 0.1 in at least one of the two time points (secondary Y-axis).

Data set 3 h mapped into 185 pathways with 32 meeting ratio of ≥ 10% and 70 meeting a threshold of P value ≤ 0.05. Similar analysis of data set 7 h resulted in genes mapping to 176 pathways with 72 achieving a ratio ≥ 10% and 44 having P values ≤ 0.05. Common canonical pathways in both time points include molecular mechanism of cancer, glioma signaling, hepatocyte growth factor (HGF) signaling, cell cycle regulation by B-cell translocation gene (BTG) family of proteins, P53 signaling, cell cycle: G2/M DNA damage checkpoint regulation, cell cycle: G1/S checkpoint regulation, ataxia telangiectasia mutated protein (ATM) signaling, ephrine receptor signaling, and virus entry via endocytic pathways. Observed order variation of identified pathways in examined time points when sorted by their gene ratios or P values underscores the time dependent viral activity (Fig. 5).

The following analysis focuses on most relevant pathways that were identified with strong statistical support of P-values < 0.05 and ratio > 10% in either time point.

#### Molecular mechanisms of cancer (MMC)

**Figure 6:**
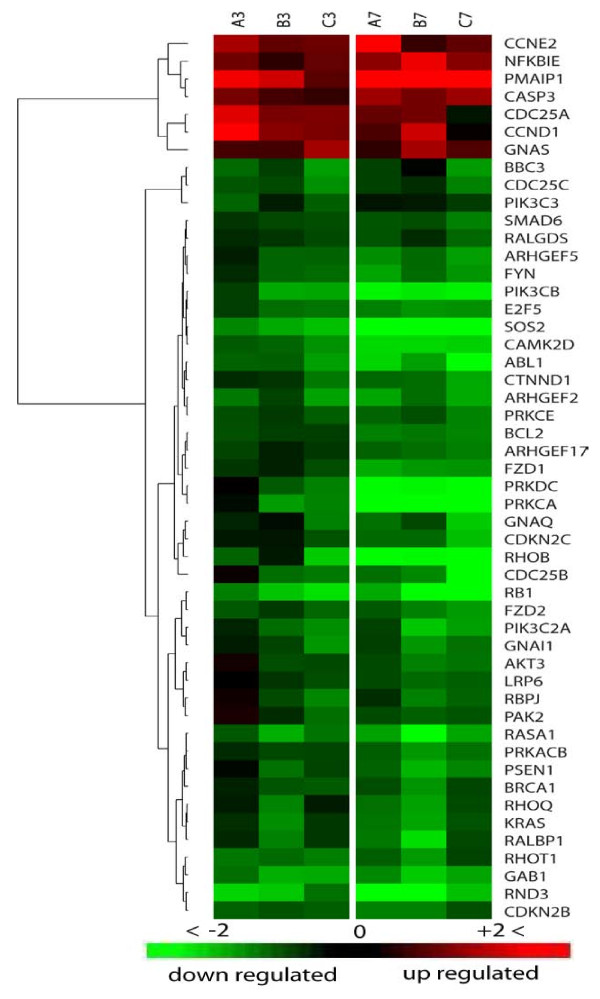
**Heat-map of differentially expressed genes in MPV infected MK2 cells that mapped to molecular mechanisms of cancer pathway**. Columns represent expression at 3 and 7 hpi in a triplicate (A, B, C). Each row represents one gene that met analysis cutoff of average fold change ≥1.8 in at least one of the two time points and P-value ≤ 0.05 in both. A gradient of green and red colors represent low and high relative fold change of gene expression to mock- infected cells. Chance for random association of listed genes at 3 or 7 hpi in this pathway is 2.82e-6 or 1.25e-5, respectively.

With 370 molecules localized to cell membrane, mitochondrion inner and outer membrane, cell cytoplasm, and cell nucleus, this pathway is considered one of the most complex known pathways. Forty-eight genes of data set 7 hpi clustered to MMC to cover 13% of total pathway molecules with P-value of 1.25e-5, making it the first pathway influenced by MPV infection. The 3 hpi data set showed lower gene ratio of 9.5% but with a tighter correlation at lower P-value of 2.82e-6 (Fig. 6).

MMC pathway can be triggered by diverse stimuli including hormones, growth factors, cytokines, and oncogenes. Each of these stimuli is capable of inducing a variety of biological processes relating to cancer, including cell cycle regulation and cell cycle checkpoints, which were recognized in our analysis as independent pathways, and therefore will be discussed separately. The majority of the genes specific to cancer mechanisms pathway are involved in apoptosis regulation. Identified modulation in expression patterns of these genes suggest signaling apoptosis in infected cells, e.g., Noxa or PMAIP1 (phorbol-12-myristate-13-acetate-induced protein 1) shows more than 44-fold upregulation at 7 h PI. This molecule inactivates the anti-apoptotic molecule Bcl2 (B-cell CLL/lymphoma 2)[27-29], which regulates mitochondrial membrane potential by controlling the voltage dependant anion channel (VDAC) [30-32], consequently increasing the mitochondrial membrane permeability and the release of cytchrome C that serves as a major apoptosis inducer [33]. In addition to Bcl2 inactivation, the expression of Bcl2 gene itself exhibited a significant -2.83-fold suppression, which would enhance apoptosis signaling. Apoptosis-induced cysteine-aspartic acid peptidases (caspases) such as caspase 3, which enhance the degradation of Bcl2 [30] and trigger subprograms for cell dismantling and removal, exhibited 3.2-fold upregulation. Caspase 7, 8, and 10 expression remained unchanged, and only caspase 6 [34], which functions as a downstream enzyme in the caspase activation cascade, showed mild -1.2 and -1.62 FC at 3, 7 hpi time points, respectively. Other pro-apoptotic genes like BBC3 (BcL2 binding component 3) or PUMA [35] and p21 protein (Cdc42/Rac)-activated kinase 2 or PAK2 [36] showed similar suppression trend of -1.85 and -1.99 FC at 7 hpi, respectively.

Proteosomal degradation has been associated with apoptosis. Our results show a 4.05 FC upregulation of nuclear factor of kappa light polypeptide gene enhancer in B-cells inhibitor, epsilon NFKBIE, which mediates cytoplasmic sequestering of transcription factor and acts as a transcription repressor. The effect of NFKBIE upregulation on apoptosis is unclear because it was reported to exhibit a cell line dependence [37].

Cell division cycle 25 homolog A, B, and C expression showed a remarkable regulation; e.g., Cdc25, which plays a key role in promoting progress through S phase [38], showed 1.7 FC, while Cdc 25 B and C, which regulate and promote entry into mitosis [39,40], exhibited -4.3 and -1.92 FC, respectively. Similarly, cyclin -dependent kinase regulators, cyclin E2 and cyclin D1, which are strongly tied to a variety of cancers [41-43], showed upregulation of 3.2 and 2.2 FC, respectively.

Other cell cycle regulators that have overlapping functions with apoptosis mechanisms showed sharp downregulation. This include members of the p21 RAS group such as KRAS (v-Ki-ras2 Kirsten rat sarcoma viral oncogene homolog) and Ras homolog or the RHO GTPase protein group including RHOB, RHOQ, RHOT1, and RND3 (Fig. 6).

#### Ephrine signaling pathway (ESP)

**Figure 7:**
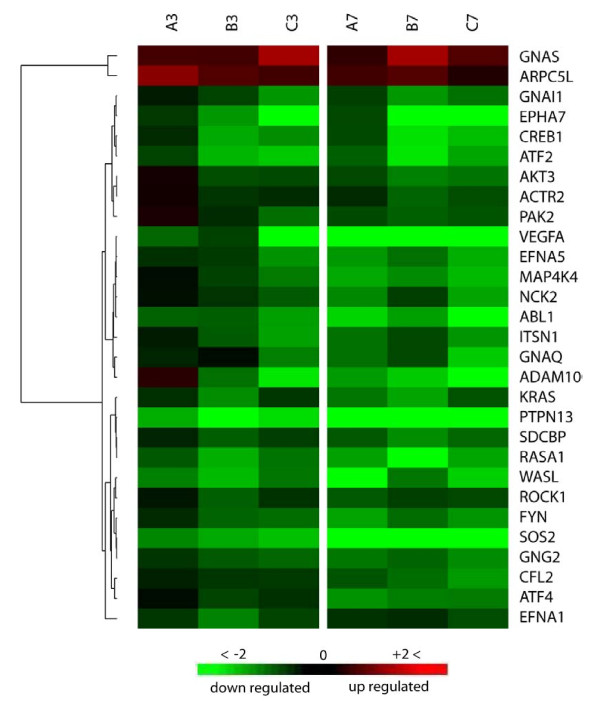
**Cluster Analysis of expression profile for genes of ephrin receptor signaling pathway influenced by MPV infection**. Columns of the heat-map represent expression of genes transcripts at 3 and 7 hpi in a triplicate (A, B, C). Each row represents one gene that met the analysis parameters of ≥1.8 average fold change cutoff in at least one of the two time points and P-value ≤ 0.05 in both. Gradient green and red color represents low and high relative expression fold change to mock infected cells, respectively. Chance for random association of listed genes at 3 or 7 hpi with this pathway is 5.95e-5 or 1.37e-4, respectively.

The largest group of receptor tyrosine kinases (RTKs), Eph receptor family and their ligands, Ephrines, were identified among the main pathways that were influenced by MPV infection. Genes in the 7 hpi data set were enriched to ESP at ratio of 13.8% of the total 195 pathway genes with a P-value of 1.37e-4 (Fig. 5). The percentage of pathway genes regulated by infection was lower during the 3 hpi time point, reaching only a ratio of 10.3% with P-value of 5.95e-5, and ranking fourth among influenced pathways by MPV infection. Transmembrane receptors Eph A class, which contains eight members of ephrine receptors and represent one of two classes forming the entire receptors family [44], exhibited significant suppression associated with downregulation of key kinases and protein groups implicated in cell morphology, cell proliferation, and cell repulsion. Of these molecules, we mention FYN oncogene, RAS p21 protein activator (GTPase activating protein RAS-GAP) 1, p21 protein (Cdc42/Rac)-activated kinase (PAK), and Rho-associated, coiled-coil containing protein kinase 1 (member of ROCK group) (Fig. 7). Interestingly, the other class of ephrines family, Eph B, didn't show any significant change in its expression in both time points. However many molecules downstream of Eph B in the signal cascade were downregulated, including intersectin 1 (SH3 domain protein), c-abl oncogene 1-RTK (ABL), and Wiskott-Aldrich syndrome-like (WASL), cofilin 2 (CFL2), actin-related protein 2 homolog (ARP2), and mitogen-activated protein kinase 4 (NcK) (Fig. 7).

Two proteins were up-regulated in this pathway, ARPC5L (actin-related protein 2/3 complex, subunit 5-like), which functions as an actin-binding protein and is involved in regulation of actin filament polymerization [45] and GNAS complex locus, which is a member of G-protein alpha subunit that has an indirect effect on signaling of ephrine pathway. The importance of this pathway is due to its involvement in the modulation of integrin activity, actin reorganization, and cellular dynamics, which are all central to viral motility, exit and invasion of proximal cells.

#### Ataxia telangiectasia mutated protein signaling pathway

**Figure 8:**
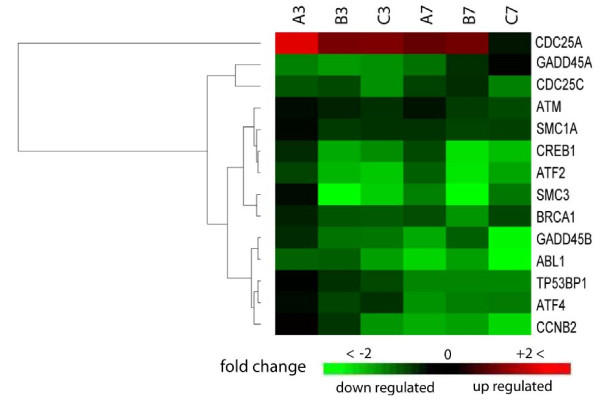
**Cluster analysis of expression profile for genes of ataxia telangiectasia mutated (ATM) pathway**. Columns of the heat-map represent expression of genes transcripts at 3 and 7 hpi time points in a triplicate (A, B, C). Each row represents one gene that met the analysis criteria of ≥1.8 average fold change cutoff in at least one of the two time points and P-value ≤ 0.05 in both. Gradient green and red color represents low and high relative expression fold change to mock infected cells, respectively. Chance for random association of listed genes at 3 or 7 hpi with this pathway is 1.87e-3 or 4.53e-3, respectively.

Ataxia telangiectasia mutated protein (ATM) is a cell kinase that phosphorylates a wide range of substrates in many pathways regulating cell cycle, DNA repair, apoptosis, and cell survival. Data sets analysis showed mild ATM downregulation of 1.3 and 1.5 FC in time points 3 and 7 hpi, respectively. Nonetheless, ATM pathway was recognized in our analysis as one of the major pathways influenced by MPV infection. Out of the 52 genes comprised in the ATM pathway, eight genes exhibited significant expression modulation with P-value of 1.87e-3 at 3 hpi. The number of genes regulated at 7 hpi increased to 10 genes, resembling 19.2% of the total pathway components with P-value of 4.53e-3. The observed suppression of ATM results in weaker signaling of the pathway and consequently reduces response to DNA damage and routine DNA repair, additionally, it disables a major apoptosis activation process. The MPV infection influence on the pathway is exacerbated further by the downregulation of many vital ATM substrates needed for proper ATM signaling. This includes activating transcription factor 2 (ATF2), c-abl oncogene 1 receptor tyrosine kinase (c-Abl), and breast cancer 1 (BRCA1) (Fig. 8).

#### Pathways involved in cell cycle regulation

**Figure 9:**
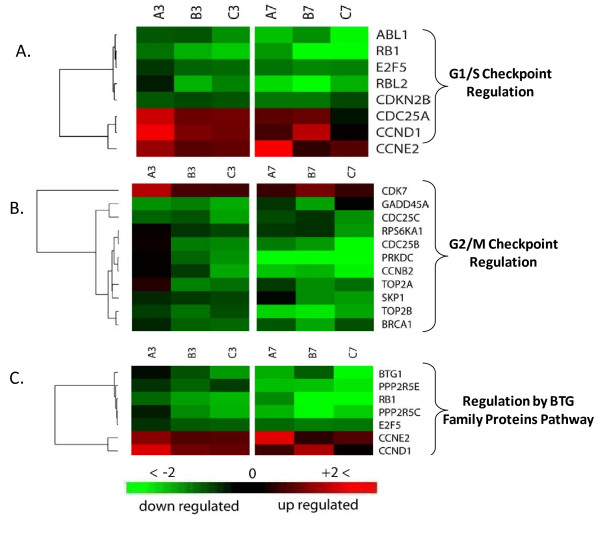
**Cluster analysis of differentially- expressed genes involved in cell cycle regulation**. Heat-map of gene expression levels at 3 and 7 hpi in triplicate (A, B, C). Folds of change in gene expression represented as a gradient of green and red color for low- and high-expression intensity, respectively. Each row represents one gene that met analysis cutoff of average fold change ≥1.8 in at least one of the two time points and P-value ≤ 0.05 in both. Genes fell in three cell cycle pathways with some genes functioning in more than one pathway **(A) **cell cycle: G1/S checkpoint regulation pathway **(B) **cell cycle: G2/M checkpoint regulation pathway **(C) **cell cycle regulation by BTG family proteins pathway.

A significant proportion of the genes in data sets 3 and 7 hpi were enriched in three major cell cycle regulation pathways. Out of the 36 molecules found in cell-cycle regulation by BTG family pathway, seven molecules were found in each of the 3 and 7 hpi data sets with P-values of 8.15e-4 at 3 hpi and 1.48e-2 at 7 hpi indicating the involvement of this pathway in early time point. Essential genes to pathway signaling and G1 arrest were severely suppressed (Figure. 9A). Retinoblastoma 1 (Rb) and B-cell translocation gene-1 (BTG1) were downregulated in both time points and reached -9.26 and -15.86 FC by 7 hpi. Furthermore, cyclin D1 and cyclin E exhibited 4.18- and 2.77-fold increase at 3 hpi respectively, and remained upregulated to similar levels at 7 hpi time point. The products of these two genes function as regulators that inactivate the growth suppression activity of Rb indirectly by promoting CDK kinases [46-48]. Phosphorylation of Rb by CDK kinase inactivates its function, leading to the release of E2F and cell cycle progression [49,50]. The net outcome of this pattern of gene expression in this pathway is cell cycle release from potential arrest in G1 phase and block of BTG1-induced apoptosis.

The second pathway contributing to cell cycle regulation that was influenced by infection is the cell cycle G1/S checkpoint. The pathway consists of 59 molecules from which eight molecules or 13.6% showed significant regulation in both data sets (Fig. 9B). The calculated P-values for random implication of this pathway were 2.71e-3 and 5.02e-2 for the 3 and 7 hpi, respectively. Similar to regulation by the BTG protein family pathway, phosphorylation of retinoblastoma Rb by CDK2 and CDK4/6 induces the release of transcription factor E2F from an inhibitory complex, which in turn promotes the transcription of necessary molecules for G1/S phase progression [50-52]. Cell-division-cycle 25 homolog A (Cdc25A), which activates CDK2 and CDK4/6 [53-55], showed strong upregulation suggesting enhancement of kinase activity that promotes G1/S phase progression. Transforming growth factor β (TGFβ) that stimulates G1/S check point exhibited almost steady suppression of -1.48 and -1.52 FC in both time points [56,57]. Another mechanism of G1/S checkpoint control is degradation of cyclin D and E after activation by glycogen synthase kinase β (GSKβ) [58,59]. Both time points show upregulation of cyclin D and E expression with no change in GSKβ expression despite mild reduction in TGFβ. This multigene expression pattern in both data sets points to destabilized G1/M control checkpoint and enhancement of cell cycle progression through this phase, especially in the early time point as suggested by lower P-value and greater gene regulation.

The third cell-cycle-related pathway identified in our analysis was the G2/M DNA damage checkpoint regulation pathway. About 14% of the total 43 molecules comprised in this pathway exhibited significant expression modulation upon infection at time point 3 hpi with P-value of 5.02e-2. Time point 7 hpi showed regulation of 20.9% of total pathway with P-value of 1.19e-3, implying a wider pathway role during later stage of infection. Progression of cell cycle into mitosis requires the activation of cell-division cycle-2 (Cdc2) by the rapid dephosphorylation of its tyrosine 15 (Tyr-15) via action of Cdc25 and subsequent binding to cyclin B [60,61]. Damaged DNA during G2 phase triggers ATM and ataxia telangiectasia mutated and rad3-related (ATR) [62-64], which in turn activates two key kinases, Chk2 and Chk1[65,66]. The latter kinase inhibits Cdc25B and Cdc25C which function as a phosphorylase that activate Cdc2 before binding to cyclin B and entry into mitosis [67]. Our results showed that ATR/ATM, and Chk1, which activate sequentially in response to DNA abnormalities, exhibited no or only a subtle change in their expression in both time points, suggesting retention of their Cdc25B/C inhibitory effect as in non-infected cells. Three essential molecules needed for entry into mitosis exhibited substantial downregulation; Cdc25B/C and cyclin B exhibited -4.33, -1.91, -4.48 FC at 7 hpi and -1.81, -2.27, -1.71 FC at 3 hpi respectively. The observed regulation of these genes would intensify this checkpoint signaling leading to a further delay in entry into mitosis (Fig. 9C).

## Discussion

Apoptosis is a natural controlled cell death mechanism triggered by diverse stimuli to maintain tissue homeostasis and eliminate abnormal or infected cells [68]. Because apoptosis represents an important part of antiviral host response, poxviruses developed numerous ways to target it and disrupt its function [69]. Diverse anti-apoptotic viral strategies are identified in different poxviruses, e.g., *Molluscum contagiosum *virus (MCV) inhibits caspase-8 by expressing MC159 gene, which encodes a protein that binds procaspase-8 and Fas-associated death domain, thereby inhibiting death receptor-induced apoptosis mediated by Fas, TNF, or TRAIL receptors [70]. MC066 gene encodes a protein with glutathione peroxidase-like function to convert oxygen-reactive species to neutral molecules, hence preventing apoptosis triggered by increased oxidative stress associated with infection [71]. Little is known about apoptosis in cells infected with MPV, but related orthopoxviruses exhibit clear anti-apoptotic functions. Cowpox virus for instance expresses a protein that can block apoptosis in multiple ways. One of the most potent anti-apoptotic proteins is the cytokine response modifier A (CrmA) which inhibits caspase-8, caspase-10, and blocks garnzyme B-mediated apoptosis [72,73]. Similarly, *Vaccinia *viruses use SPI-2 family protein member of serine protease inhibitors (serpins) encoded by B13R gene to block apoptosis induced by death receptors [74]. The same virus inhibits apoptosis induced by RNA-dependent protein kinase (PKR) using specific PKR inhibitors encoded by E3L and K3L genes [75], and apoptosis induced by loss of mitochondria outer membrane potential by expression of F1L gene [76].

The presence of many viral proteins that block apoptosis at multiple points suggests that apoptosis is detrimental to viral survival. However, the observed downregulation of Bcl-2, PUMA, and PAK2 and upregulation of NOXA and caspase-3 with the negligible change in other apoptotic genes in our data is more consistent with apoptosis induction. Although many pathogens and viruses other than poxviruses are reported to promote apoptosis [77], it is unlikely that MPV will differ from other poxviruses in their common anti-apoptotic trend, and the observed divergence between the regulation of apoptosis-specific genes in MPV-infected cells and overall anti-apoptotic outcome seen in other poxvirus-infected cells suggests an anti-apoptotic viral mechanism that functions downstream of apoptosis induction in the host. MPV genes involved in blocking apoptosis remain unknown, but our data suggests the presence of an ortholog(s) of *Vaccinia *virus (F1L) gene in MPV, which acts directly on the mitochondria in Bcl-2 like manner.

Double-stranded DNA mammalian viruses have large genomes reaching 289 Kbp or few hundreds of micrometers in length as in the case of fowlpox virus [78]. Compacting the genome is an indispensible biological task due to the rich negative charge and large size of DNA molecules. In eukaryotes, this was solved by wrapping DNA around a heterooctameric molecule composed of four dimerized, positively charged core histones to form nucleosomes. Further stability is brought about by linker histones that bind DNA laterally. Nucleosomes, in a nucleofilaments form or in a form of higher-order-structure assemblies, chromatin, play an essential role in the regulation of gene expression and chromatin remodeling via acetylation and deacetylation of the N-terminal histone tails protruding from nucleosomes. Viruses exhibit similar architecture in their DNA compaction. Staining vaccinia-infected cells with osmium ammine-SO_2 _revealed areas with concentrated viral DNA in two predominant DNA configurations varying depending on the level of viral synthetic activities. Encapsidated genomes exhibited a nucleosome structure like those observed in resting eukaryotes, which was in agreement with biochemical data showing the supercoiled organization of nucleoproteins extracted from Vaccinia viruses [79]. On the other hand, like active cellular chromatin, non-encapsidated viral genomes exhibited extended DNA features [80]. Similar variation in DNA density was observed in Herpes simplex viruses (HSV), another double stranded DNA virus, with two well described configurations of euchromatin or heterochromatin that correlated strongly with lytic or latent HSV infection stages, respectively. Generally, little is known about chromatin potential in poxviruses as an antiviral mechanism. Our results showed an interesting downregulation of five essential histone expression regulation factors and five enzymes regulating chromatin dynamics in MPV-infected cells. It is unclear if these results are part of the host response, which include chromatin-mediated silencing of the viral genome and activation of DNA damage [81], or part of the viral strategies to take over its host. However, our observation predicts an important role for histone expression, histone posttranslational modification, and dynamic exchanges of chromatin in host-poxvirus interactions. Recent work suggested a role for the viral A32L gene of *Vaccinia *virus in DNA packaging based on sequence similarities with the product of gene I of filamentous single-stranded DNA bacteriophages and the Iva2 gene of adenoviruses. Both of these genes are ATPases involved in DNA packaging. Additional *Vaccinia *genes that map to I6 or I1 telomere-binding proteins are believed to play roles in DNA packaging, because mutants of VACV in either of these two genes fail to exhibit normal DNA packaging at different morphogenesis stages. The sharp upregulation we observed in three out the four core histones, and the striking similarities in DNA compaction architecture observed in eukaryotes and some viruses, with absence of known poxvirus proteins that exhibit histone-like properties makes it tempting to hypothesize a role of host cell histones in viral DNA compaction and nucleosome formation, especially that similar involvement was described recently in simian virus 40 (SV40) DNA compaction [82]

Our analysis identified ephrin receptor pathway (ERP) as a major influenced pathway in infected cells. This might be due to either increased cell to cell communication by signaling through this receptor tyrosine kinases (RTK) family in response to infection, or to the presence of many pleiotropic genes that are found in ERP, and simultaneously have essential roles in cytoskeleton reorganization or actin polymerization. Intracellular viral motility and morphogenesis of Vaccinia virus into a cell-associated enveloped virion (CEV) and extracellular enveloped virions (EEV) forms are shown to be driven by interactions of host microtubules and *Vaccinia *A27L, A17L and A14L genes. Furthermore, egress of *Vaccinia *particles in EEV form and direct cell-to-cell virus dissemination is propelled by actin tail formation, which involves the interactions of *Vaccinia *transport genes, including A36R, F12L, and host proteins such as Src family kinases (SFK), Nck, WIP, N-WASP, Arp 2/3 to promote actin filaments nucleation. Actin polymerization produces microvilli at cell surface that lift CEV and project it on adjacent cells to finally deliver the virus with minimal exposure to host immune system. Our results confirm regulation of many principal signaling components involved in actin cytoskeletal dynamics, and introduce additional infection regulated genes with functions related to microtubules signaling. This includes intersectin 1 (SH3 domain protein) gene, which encodes a cytoplasmic membrane-associated protein that indirectly coordinates endocytic membrane traffic with the actin assembly machinery, Rho-effector ROCK1 serve a number of key cellular functions, such as morphological differentiation and cell motility which are closely associated with changes in cytoskeletal dynamics [83]. Additionally, RAS p21 protein activator (GTPase activating protein) [84], v-Ki-ras2 Kirsten rat sarcoma viral oncogene homolog [85] and SOS2 [86] are crucial genes in polymerization of actin filaments and cytoskeleton reorganization.

Ion channels represent an intriguing and novel class of genes that were impacted by MPV infection. We identified 10 genes encoding nine ion channels and a transporter that underwent increasing suppression during infection. Most of these channels localize to cell membrane, and collectively contribute to transport of all essential ions involved in maintenance of cell membrane potential and osmolarity homeostasis. While mechanisms of transport modulation have been described previously, as in the indirect consequences of Ras, Rho, and Rab small GTPases regulation [87], its effect on viral infections and global cell biology remain unclear except for a recent report describing the interaction of myxoma poxvirus protein M11L with mitochondrial permeability transition pore and its role in delaying apoptosis in host cells [88]. The downregulation trend of channel expression identified here pose many intriguing questions, especially in the light of evolving evidence in support of ion channels role in virus release [89] and infected cells rupture [90].

Progression of the cell cycle is tightly regulated process with many redundant checkpoints that ensure proper transition across cell cycle phases. Our results showed significant modulation in the expression of many genes that play essential roles in cell cycle regulation, which led to the identification of ATM signaling, G2/M DNA damage checkpoint, regulation by BTG family protein, and G1/S checkpoint as major influenced pathways during MPV infection. A core cell cycle regulation gene, Cdc25 kinase, has three essential homologs Cdc 25A/B/C that exhibited significant regulation upon MPV infection. While Cdc25B/C showed downregulation favoring cell arrest in G2 phase, Cdc2A exhibited upregulation, favoring S phase progression. The impact of this mode of cell cycle regulation on viral infection remains unknown. However, cell arrest in G2 phase was described in other viral infections including human immunodeficiency virus (HIV) and was found to be mainly mediated by viral protein R (Vpr) [91-93]. While many of the G2/M DNA damage checkpoint pathway genes are known to be modulated during HIV infection, Vpr seems to induce cell arrest by molecular mechanisms other than the classic DNA check point [94]. Recently, evidence supporting a role for PP2A in Vpr-induced arrest has emerged, and was substantiated further by other studies in support of PP2A being a common target during infection with other viruses, including simian virus 40 (SV40), polyoma virus, human T lymphotrophic retrovirus and adenovirus [95]. Our results showed significant downregulation in two PP2A isoforms, regulatory subunit B' gamma isoform (PPP2R5C) and protein phosphatase 2, regulatory subunit B' epsilon isoform (PPP2R5E), suggesting that the induction mechanisms of G2 arrest in MPV infection might be similar to those observed in other viruses. Because genetically diverse viruses seem to induce the same G2 arrest response in different infected cells, it is likely that this response has an important function and might be part of antiviral host defenses. While some of the viral genes eliciting this response are being identified as Vpr in HIV, and E4 of F4 and HTLV tax protein in adenoviruses, the MPV gene inducing this response remains unknown. Other important genes in cell cycle regulation showing expression favoring progression of cell cycle and arrest only in G2 phase include Rb, E2F, cyclins, BTG1, and BRCA1.

In this study we combined microarray with data mining and statistical analysis to identify important interfaces of host-pathogen interaction. Our results aligned nicely with previous reports carried out using viruses from the same or different genus, and provided new set of genes that play important roles in MPV infection. Further work is warranted to validate and examine the potential of these genes in antiviral therapies.

## Summary

Using microarrays, we studied MPV-induced changes in gene expression of *Macaca mulatta *kidney epithelial cells to identify major host-virus interaction interfaces. Infection stimulated a marked modulation in some 2,702 host genes representing ≈ 5.7% of total interrogated host transcripts. The majority of genes (89.08%) underwent 1.5-folds downregulation or more. While downregulated genes exhibited a steady trend during the study, upregulated genes showed more time-dependent regulation intensity. Further data analysis showed that regulated genes cluster into distinctive functional classes, canonical pathways and networks that can be linked to established viral biogenesis. Our results introduce a set of host genes and novel pathways for further evaluation as targets for potential use in developing new antiviral therapies.

## Abbreviations

CITED2: Cbp/p300-interacting transactivator, with Glu/Asp-rich carboxy-terminal domain, 2; NCOA3: nuclear receptor coactivator 3; CREB: cAMP responsive element binding protein 1;YY1: YY1 transcription factor; HDAC2: histone deacetylase 2; FBXO11: F-box protein 11; PRMT3: protein arginine methyltransferase 3; MYST2: MYST histone acetyltransferase 2; MYCBP2: MYC binding protein 2; RARS2: arginyl-tRNA synthetase 2, mitochondrial; NOXA: NADPH oxidase activator; Bcl2: B-cell CLL/lymphoma 2; BBC3 or PUMA: BCL2 binding component 3; PAK2: p21 protein (Cdc42/Rac)-activated kinase 2; Cdc25: cell division cycle 25; KRAS: v-Ki-ras2 Kirsten rat sarcoma viral oncogene homolog; RHOB: ras homolog gene family, member B; RHOQ: ras homolog gene family, member Q; RHOT1: ras homolog gene family, member T1; RND3: Rho family GTPase 3; FYN: FYN oncogene related to SRC, FGR, YES; ROCK: Rho Associated Kinase, Rho kinase; ABL1/2: c-abl oncogene 1 or 2, receptor tyrosine kinase; WSAL: Wiskott-Aldrich syndrome-like; CFL2: cofilin 2; ARP2/3: actin related protein 2/3; ARPC5L: actin-related protein 2/3 complex, subunit 5-like; GNAS: GNAS complex locus; ATF2: activating transcription factor 2; c-Abl: c-abl oncogene 1 receptor tyrosine kinase; BRCA1: breast cancer 1; Rb1: Retinoblastoma 1; BTG1: B-cell translocation gene-1; CDK: cyclin-dependent kinase; TGFβ: Transforming growth factor β; GSKβ: glycogen synthase kinase β; ATR: ataxia telangiectasia mutated and rad3-related; ChK: choline kinase; TRAIL or TNFSF10: tumor necrosis factor (ligand) superfamily, member 10; FAS: Fas (TNF receptor superfamily, member 6); CrmA: cytokine response modifier A; SFK: Src family kinases; SOS2; son of sevenless homolog 2; PP2A: Protein Phosphatase Type2a; HTLV: human T-cell leukemia virus

## Competing interests

The authors declare that they have no competing interests.

## Authors' contributions

AA was responsible for design, conduct, and completion of this work, as well as for data analysis and writing of this manuscript. RH and MJ were instrumental in data processing and statistical analysis of microarray data. JH and MA contributed to microarray data validation using RT PCR. SI was the Principal Investigator and is primarily responsible for all aspects of the funding, research design, interpretation, and writing of this manuscript. All authors read and approved the final manuscript.
